# 4,4′-Dimeth­oxybi­phenyl-3,3′-di­car­box­ylic acid

**DOI:** 10.1107/S1600536814008599

**Published:** 2014-04-30

**Authors:** Fredrik Lundvall, David Stephen Wragg, Pascal D. C. Dietzel, Helmer Fjellvåg

**Affiliations:** a Centre for Materials Science and Nanotechnology, Department of Chemistry, University of Oslo, PO Box 1126, 0315 Oslo, Norway; b inGAP National Centre of Research-based Innovation, Department of Chemistry, University of Oslo, PO Box 1126, 0315 Oslo, Norway; c Department of Chemistry, University of Bergen, PO Box 7803, 5020 Bergen, Norway

## Abstract

The title compound, C_16_H_14_O_6_, was recrystallized under solvothermal conditions. The mol­ecules are located on inversion centres, with one complete mol­ecule generated from the asymmetric unit by inversion. There are intra­molecular O—H⋯O hydrogen bonds involving the carb­oxy­lic acid group and the O atom of the adjacent meth­oxy group. In the crystal, mol­ecules are linked *via* O—H⋯O hydrogen bonds, forming chains propagating along [100]. The chains are linked *via* C—H⋯O hydrogen bonds, forming sheets parallel to (001).

## Related literature   

For the synthesis, see Wang *et al.* (2009 ▶).
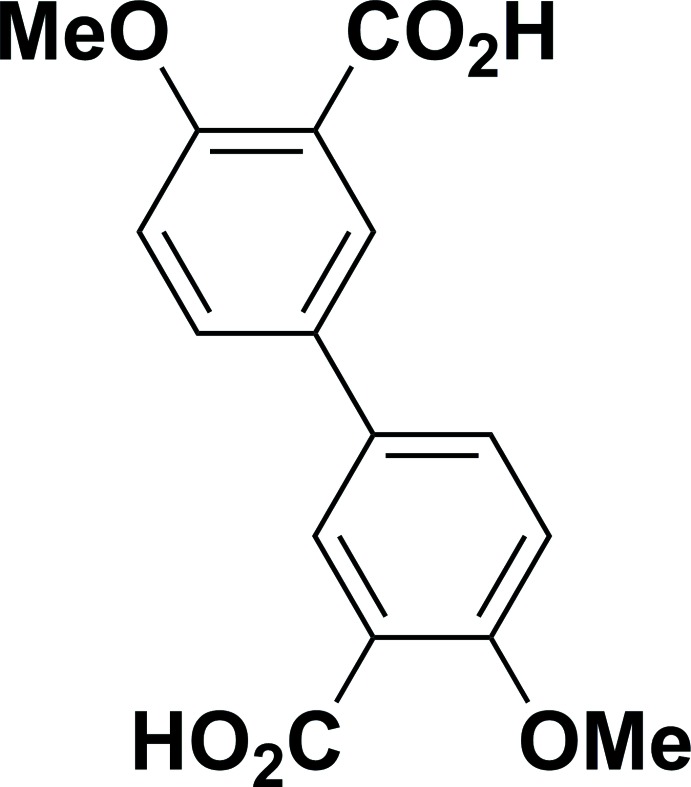


## Experimental   

### 

#### Crystal data  

C_16_H_14_O_6_
*M* *_r_* = 302.27
Orthorhombic, 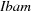

*a* = 13.138 (2) Å
*b* = 15.615 (3) Å
*c* = 6.7726 (11) Å
*V* = 1389.4 (4) Å^3^
*Z* = 4
Mo *K*α radiation
μ = 0.11 mm^−1^
*T* = 296 K
0.65 × 0.10 × 0.09 mm

#### Data collection  

Bruker APEXII CCD diffractometer
Absorption correction: multi-scan (*SADABS*; Sheldrick, 1996 ▶) *T*_min_ = 0.931, *T*_max_ = 0.990
5188 measured reflections
779 independent reflections
689 reflections with *I* > 2σ(*I*)
*R*_int_ = 0.018

#### Refinement  

*R*[*F* ^2^ > 2σ(*F* ^2^)] = 0.046
*wR*(*F* ^2^) = 0.136
*S* = 1.12
779 reflections
67 parameters
H-atom parameters constrained
Δρ_max_ = 0.24 e Å^−3^
Δρ_min_ = −0.13 e Å^−3^

### 

Data collection: *APEX2* (Bruker, 2007 ▶); cell refinement: *SAINT* (Bruker, 2007 ▶); data reduction: *SAINT*; program(s) used to solve structure: *SIR92* (Altomare *et al.*, 1994 ▶); program(s) used to refine structure: *SHELXL97* (Sheldrick, 2008 ▶); molecular graphics: *DIAMOND* (Brandenburg, 2004 ▶) and *ChemBioDraw Ultra* (CambridgeSoft, 2009 ▶); software used to prepare material for publication: *publCIF* (Westrip, 2010 ▶) and *WinGX* (Farrugia, 2012 ▶).

## Supplementary Material

Crystal structure: contains datablock(s) I, New_Global_Publ_Block. DOI: 10.1107/S1600536814008599/qm2105sup1.cif

Structure factors: contains datablock(s) I. DOI: 10.1107/S1600536814008599/qm2105Isup2.hkl

Supporting information file. DOI: 10.1107/S1600536814008599/qm2105Isup3.cml

CCDC reference: 997556

Additional supporting information:  crystallographic information; 3D view; checkCIF report

## Figures and Tables

**Table 1:** Hydrogen-bond geometry (Å, °)

*D*—H⋯*A*	*D*—H	H⋯*A*	*D*⋯*A*	*D*—H⋯*A*
O1—H1⋯O3	0.82	1.85	2.545 (2)	141
O1—H1⋯O1^i^	0.82	2.42	2.816 (3)	111
C5—H5⋯O2^ii^	0.93	2.41	3.341 (2)	175

Symmetry codes: (i) 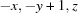; (ii) 
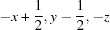
.

**Fig. 1.**
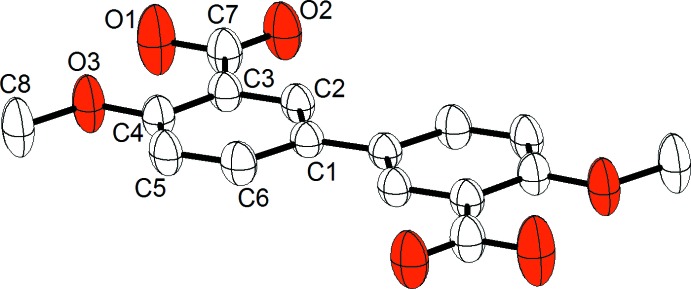
[One molecular unit of the title compound with atom labels and 50% probability displacement ellipsoids. Hydrogen atoms are omitted for clarity.]

**Fig. 2.**
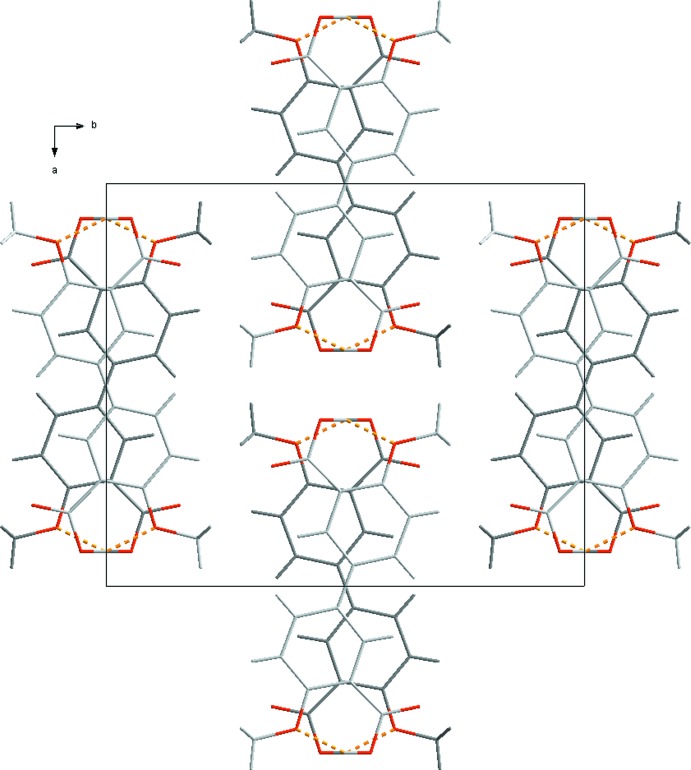
[Packing diagram of the title compound viewed along the c axis. Hydrogen bonds are indicated by dashed lines.]

**Fig. 3.**
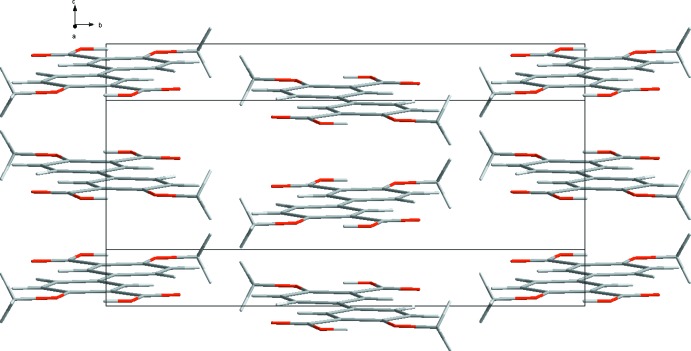
[Packing diagram of the title compound viewed close to the a axis.]

## supplementary crystallographic information

### 1. Comment

As a part of a larger project, the title compound was synthesized for use as an organic linker in MOFs (Metal-Organic Frameworks).

The title compound has previously been reported (Wang *et al.*, 2009) as an intermediate in the synthesis of an arylamide. The crystal structure was however not reported in this publication.

The structure of the title compound C_16_H_14_O_6_, has an orthorhombic Ibam symmetry. The asymmetric unit of the compound contains one half of the molecule, with the complete molecule being generated by an inversion centre. The two benzene rings appear as planar relative to each other and the carboxylic acid groups are located in a *trans* fashion with regards to the bond between the benzene rings. Biphenyl compounds commonly feature a torsion angle between the benzene rings, and the relatively large thermal parameters of the atoms furthest away from the molecular axis could indicate that a small torsion angle is present. Thus, the apparent planar configuration of the benzene rings might be considered a crystallographical artifact. Intramolecular hydrogen bonding between H1 and O3 directs the orientation of the hydroxyl group. Intermolecular hydrogen bonds between the O1 oxygen atoms of neighbouring molecules arrange the molecules in one-dimensional zigzag chains. These chains are further packed to form two-dimensional layers stabilized by hydrogen bonds between the carbonyl oxygen (O2) and one aromatic hydrogen (H5). It is worth noting that the carboxylic acid dimer motif thus is absent in this structure. The molecules are ordered along the *c* axis in a staggered motif with an intermolecular distance equal to one half of the *c* axis. This distance might indicate some weak π–π stacking interaction between the two-dimensional layers.

### 2. Experimental

The title compound was synthesized by a slightly modified version of the method used by Wang *et al.* (2009).

In the synthesis of methyl 5-iodo-2-methoxybenzoate, the reaction time was increased from 30 to 60 minutes.

In the Ullmann-coupling of 2 equivalents of methyl 5-iodo-2-methoxybenzoate to form dimethyl 4,4'-dimethoxy-3,3'-dicarboxylate, the reaction temperature was increased to 225 °C and the reaction time was set to 8 h.

In the synthesis of the title compound, dimethyl 4,4'-dimethoxy-3,3'-dicarboxylate and potassium hydroxide was stirred in a mixture of water and THF under reflux for 18 h. The mixture was concentrated under reduced pressure, washed with diethyl ether and acidified with nitric acid. The product was separated from the mixture by filtration and washed with water. The ^1^H NMR spectrum of the title compound is in good agreement with what was reported by Wang *et al.* (2009).

The title compound (151 mg, 0.5 mmol) was subjected to solvothermal conditions (H_2_O, 100 °C for 2 days) in the precence of Ca(NO_3_)_2˙_4H_2_O (118 mg, 0.5 mmol) and NaOH (40 mg, 1.0 mmol). The procedure did not yield the desired MOF, the title compound was however recrystallized into single crystals suitable for X-ray diffraction.

### 3. Refinement

The structure was refined by full-matrix least squares using *SHELXL97* (Sheldrick, 2008) as implemented in the *WinGX* suite (Farrugia, 2012). H-atoms were positioned geometrically at distances of 0.82 (OH), 0.93 (CH) and 0.96 Å (CH_3_) and refined using a riding model with *U*_iso_ (H)=1.2 *U*_eq_ (CH) and *U*_iso_ (H)=1.5 *U*_eq_ (OH and CH_3_)

### Crystal data

**Table:**

C_16_H_14_O_6_	*F*(000) = 632
*M**_r_* = 302.27	*D*_x_ = 1.445 Mg m^−^^3^
Orthorhombic, *I**b**a**m*	Mo *K*α radiation, λ = 0.71073 Å
Hall symbol: -I 2 2c	Cell parameters from 2142 reflections
*a* = 13.138 (2) Å	θ = 2.6–28.2°
*b* = 15.615 (3) Å	µ = 0.11 mm^−^^1^
*c* = 6.7726 (11) Å	*T* = 296 K
*V* = 1389.4 (4) Å^3^	Needle, yellow
*Z* = 4	0.65 × 0.10 × 0.09 mm

### Data collection

**Table:**

Bruker APEXII CCD diffractometer	779 independent reflections
Radiation source: fine-focus sealed tube	689 reflections with *I* > 2σ(*I*)
Graphite monochromator	*R*_int_ = 0.018
φ and ω scans	θ_max_ = 26.4°, θ_min_ = 2.0°
Absorption correction: multi-scan (*SADABS*; Sheldrick, 1996)	*h* = −16→15
*T*_min_ = 0.931, *T*_max_ = 0.990	*k* = −19→19
5188 measured reflections	*l* = −8→8

### Refinement

**Table:**

Refinement on *F*^2^	Primary atom site location: structure-invariant direct methods
Least-squares matrix: full	Secondary atom site location: difference Fourier map
*R*[*F*^2^ > 2σ(*F*^2^)] = 0.046	Hydrogen site location: inferred from neighbouring sites
*wR*(*F*^2^) = 0.136	H-atom parameters constrained
*S* = 1.12	*w* = 1/[σ^2^(*F*_o_^2^) + (0.0765*P*)^2^ + 0.4964*P*] where *P* = (*F*_o_^2^ + 2*F*_c_^2^)/3
779 reflections	(Δ/σ)_max_ < 0.001
67 parameters	Δρ_max_ = 0.24 e Å^−^^3^
0 restraints	Δρ_min_ = −0.13 e Å^−^^3^

### Special details

**Table:**

Geometry. All e.s.d.'s (except the e.s.d. in the dihedral angle between two l.s. planes) are estimated using the full covariance matrix. The cell e.s.d.'s are taken into account individually in the estimation of e.s.d.'s in distances, angles and torsion angles; correlations between e.s.d.'s in cell parameters are only used when they are defined by crystal symmetry. An approximate (isotropic) treatment of cell e.s.d.'s is used for estimating e.s.d.'s involving l.s. planes.
Refinement. Refinement of *F*^2^ against ALL reflections. The weighted *R*-factor *wR* and goodness of fit *S* are based on *F*^2^, conventional *R*-factors *R* are based on *F*, with *F* set to zero for negative *F*^2^. The threshold expression of *F*^2^ > σ(*F*^2^) is used only for calculating *R*-factors(gt) *etc*. and is not relevant to the choice of reflections for refinement. *R*-factors based on *F*^2^ are statistically about twice as large as those based on *F*, and *R*- factors based on ALL data will be even larger.

### Fractional atomic coordinates and isotropic or equivalent isotropic displacement parameters (Å^2^)

**Table:**

	*x*	*y*	*z*	*U*_iso_*/*U*_eq_	Occ. (<1)
C1	0.44707 (14)	0.48358 (10)	0.0000	0.0371 (5)	
C2	0.36404 (15)	0.53875 (11)	0.0000	0.0386 (5)	
H2	0.3761	0.5974	0.0000	0.046*	
C3	0.26411 (14)	0.51030 (12)	0.0000	0.0396 (5)	
C4	0.24524 (16)	0.42222 (12)	0.0000	0.0428 (5)	
C5	0.32608 (16)	0.36538 (12)	0.0000	0.0478 (6)	
H5	0.3141	0.3067	0.0000	0.057*	
C6	0.42446 (16)	0.39603 (12)	0.0000	0.0457 (6)	
H6	0.4779	0.3570	0.0000	0.055*	
C7	0.18295 (17)	0.57787 (13)	0.0000	0.0492 (6)	
C8	0.1198 (2)	0.30898 (14)	0.0000	0.0694 (8)	
H8A	0.0471	0.3030	0.0000	0.104*	
H8B	0.1474	0.2821	0.1157	0.104*	0.50
H8C	0.1474	0.2821	−0.1157	0.104*	0.50
O1	0.08706 (12)	0.55256 (10)	0.0000	0.0868 (8)	
H1	0.0839	0.5026	−0.0370	0.130*	0.50
O2	0.20123 (13)	0.65265 (9)	0.0000	0.0658 (6)	
O3	0.14575 (12)	0.39725 (9)	0.0000	0.0629 (6)	

### Atomic displacement parameters (Å^2^)

**Table:**

	*U*^11^	*U*^22^	*U*^33^	*U*^12^	*U*^13^	*U*^23^
C1	0.0400 (11)	0.0292 (10)	0.0422 (10)	−0.0009 (7)	0.000	0.000
C2	0.0414 (11)	0.0260 (8)	0.0484 (11)	−0.0003 (7)	0.000	0.000
C3	0.0390 (10)	0.0284 (10)	0.0515 (11)	0.0009 (8)	0.000	0.000
C4	0.0365 (11)	0.0331 (10)	0.0586 (12)	−0.0037 (7)	0.000	0.000
C5	0.0461 (12)	0.0253 (9)	0.0718 (15)	−0.0020 (8)	0.000	0.000
C6	0.0415 (11)	0.0281 (10)	0.0675 (14)	0.0039 (8)	0.000	0.000
C7	0.0403 (12)	0.0308 (10)	0.0765 (15)	0.0016 (8)	0.000	0.000
C8	0.0497 (13)	0.0349 (11)	0.124 (2)	−0.0119 (10)	0.000	0.000
O1	0.0360 (9)	0.0414 (9)	0.183 (2)	0.0040 (7)	0.000	0.000
O2	0.0488 (9)	0.0284 (8)	0.1201 (16)	0.0036 (6)	0.000	0.000
O3	0.0399 (9)	0.0307 (8)	0.1181 (15)	−0.0046 (6)	0.000	0.000

### Geometric parameters (Å, º)

**Table:**

C1—C2	1.390 (3)	C5—H5	0.9300
C1—C6	1.399 (2)	C6—H6	0.9300
C1—C1^i^	1.482 (4)	C7—O2	1.192 (2)
C2—C3	1.386 (3)	C7—O1	1.320 (3)
C2—H2	0.9300	C8—O3	1.420 (2)
C3—C4	1.398 (3)	C8—H8A	0.9600
C3—C7	1.500 (3)	C8—H8B	0.9600
C4—O3	1.364 (3)	C8—H8C	0.9600
C4—C5	1.384 (3)	O1—H1	0.8200
C5—C6	1.378 (3)		
			
C2—C1—C6	116.04 (18)	C5—C6—C1	122.59 (18)
C2—C1—C1^i^	121.45 (19)	C5—C6—H6	118.7
C6—C1—C1^i^	122.5 (2)	C1—C6—H6	118.7
C3—C2—C1	123.00 (17)	O2—C7—O1	119.04 (19)
C3—C2—H2	118.5	O2—C7—C3	123.1 (2)
C1—C2—H2	118.5	O1—C7—C3	117.88 (17)
C2—C3—C4	118.92 (18)	O3—C8—H8A	109.5
C2—C3—C7	116.61 (17)	O3—C8—H8B	109.5
C4—C3—C7	124.48 (18)	H8A—C8—H8B	109.5
O3—C4—C5	123.50 (18)	O3—C8—H8C	109.5
O3—C4—C3	116.84 (18)	H8A—C8—H8C	109.5
C5—C4—C3	119.67 (19)	H8B—C8—H8C	109.5
C6—C5—C4	119.79 (17)	C7—O1—H1	109.5
C6—C5—H5	120.1	C4—O3—C8	120.52 (17)
C4—C5—H5	120.1		
			
C6—C1—C2—C3	0.0	C4—C5—C6—C1	0.0
C1^i^—C1—C2—C3	180.0	C2—C1—C6—C5	0.0
C1—C2—C3—C4	0.0	C1^i^—C1—C6—C5	180.0
C1—C2—C3—C7	180.0	C2—C3—C7—O2	0.0
C2—C3—C4—O3	180.0	C4—C3—C7—O2	180.0
C7—C3—C4—O3	0.0	C2—C3—C7—O1	180.0
C2—C3—C4—C5	0.0	C4—C3—C7—O1	0.0
C7—C3—C4—C5	180.0	C5—C4—O3—C8	0.0
O3—C4—C5—C6	180.0	C3—C4—O3—C8	180.0
C3—C4—C5—C6	0.0		

Symmetry code: (i) −*x*+1, −*y*+1, −*z*.

### Hydrogen-bond geometry (Å, º)

**Table:**

*D*—H···*A*	*D*—H	H···*A*	*D*···*A*	*D*—H···*A*
O1—H1···O3	0.82	1.85	2.545 (2)	141
O1—H1···O1^ii^	0.82	2.42	2.816 (3)	111
C5—H5···O2^iii^	0.93	2.41	3.341 (2)	175

Symmetry codes: (ii) −*x*, −*y*+1, *z*; (iii) −*x*+1/2, *y*−1/2, −*z*.

## References

[bb1] Altomare, A., Cascarano, G., Giacovazzo, C., Guagliardi, A., Burla, M. C., Polidori, G. & Camalli, M. (1994). *J. Appl. Cryst.* **27**, 435.

[bb2] Brandenburg, K. (2004). *DIAMOND* Crystal Impact GbR, Bonn, Germany.

[bb3] Bruker (2007). *APEX2* and *SAINT* Bruker AXS Inc., Madison, Wisconsin, USA.

[bb4] CambridgeSoft (2009). *ChemBioDraw Ultra* CambridgeSoft Corporation, Cambridge, Massachusetts, USA.

[bb5] Farrugia, L. J. (2012). *J. Appl. Cryst.* **45**, 849–854.

[bb6] Sheldrick, G. M. (1996). *SADABS* University of Göttingen, Germany.

[bb7] Sheldrick, G. M. (2008). *Acta Cryst.* A**64**, 112–122. 10.1107/S0108767307043930 18156677

[bb8] Wang, L., Xiao, Z.-Y., Hou, J.-L., Wang, G.-T., Jiang, X.-K. & Li, Z.-T. (2009). *Tetrahedron*, **65**, 10544–10551.

[bb9] Westrip, S. P. (2010). *J. Appl. Cryst.* **43**, 920–925.

